# The Non-Coding RNA *GAS5* and Its Role in Tumor Therapy-Induced Resistance

**DOI:** 10.3390/ijms21207633

**Published:** 2020-10-15

**Authors:** George I. Lambrou, Kyriaki Hatziagapiou, Apostolos Zaravinos

**Affiliations:** 1 Choremeio Research Laboratory, First Department of Pediatrics, National and Kapodistrian University of Athens, Thivon & Levadeias 8, 11527 Goudi, Athens, Greece; khatziag@med.uoa.gr; 2 Department of Basic Medical Sciences, College of Medicine, Member of QU Health, Qatar University, 2713 Doha, Qatar

**Keywords:** *GAS5*, malignancy, proliferation, invasion, metastasis, tumor growth

## Abstract

The growth arrest-specific transcript 5 (*GAS5*) is a >200-nt lncRNA molecule that regulates several cellular functions, including proliferation, apoptosis, invasion and metastasis, across different types of human cancers. Here, we reviewed the current literature on the expression of *GAS5* in leukemia, cervical, breast, ovarian, prostate, urinary bladder, lung, gastric, colorectal, liver, osteosarcoma and brain cancers, as well as its interaction with various miRNAs and its effect on therapy-related resistance in these malignancies. The general consensus is that *GAS5* acts as a tumor suppressor across different tumor types and that its up-regulation results in tumor sensitization to chemotherapy or radiotherapy. *GAS5* seems to play a previously unappreciated, but significant role in tumor therapy-induced resistance.

## 1. Introduction

The massive and rapid increase in the amount of human genome-scale DNA sequencing and the parallel development of methods to exploit these data drive the biomedical research today in a significant transition. The three billion base pairs of human DNA do not provide information about the function of the genes, or how cells grow, divide, form organisms, how mistakes in them are reflected in diseases, and how to develop a drug. Thus, it is important to construct a catalogue of expressed or suppressed genes for each cellular function, in order to understand how each component works within living cells. The transcriptome, i.e., the genes that are transcribed into mRNA, determines the phenotype and function of each cell [1]. Thus, alterations in gene expression are highly dynamic; they drive cellular phenotypic characteristics, DNA replication and cell division, as well as how a cell responds to an extracellular stimulus or perturbation. The understanding of when, where and to what extent a gene is expressed, can elucidate the regulatory mechanisms and biological pathways that lead to, maintain or reverse multiple drug-resistance in cancer treatment. Almost twenty years ago, the non-coding RNAs (ncRNAs) were discovered [2,3] and provided a breakthrough in our understanding of the functionality of the human genome. To take advantage of the large and rapidly increasing body of genome-scale sequence information, new technologies are required to exploit this information by characterizing biological processes and by studying the synchronous expression of a high number of genes. A variety of techniques has evolved to monitor, rapidly and efficiently, the transcript abundance of all genes in an organism [4].

Thousands of genes have been identified through high throughput methodologies, and a plethora of them have been studied for their role in tumor progression, as well as therapy-induced resistance.

## 2. The Long Non-Coding RNA Repressor *GAS5*

**Figure 1:**
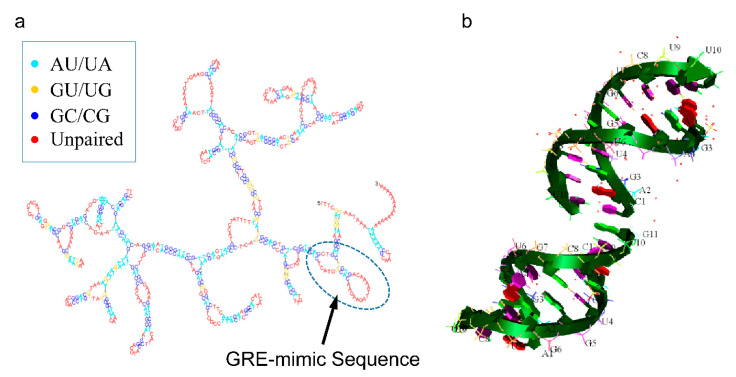
Secondary structure of the growth arrest-specific 5 (*GAS5*) RNA, showing the glucocorticoid receptor element (GRE)-mimic sequence (**a**), along with the 3D structure of the GRE-mimic sequence (**b**) (the 3D structure of the GRE-mimic was obtained from the Protein Data Bank with reference no. 4MCE [18]).

The ncRNAs are essential players in many cellular processes, from normal development to oncogenic transformation, offering an additional level of regulatory complexity in the transcription of mammalian genes [5,6,7]. They can be divided into microRNAs (miRNAs), piwi-interacting (piRNAs), small nucleolar (snoRNAs), long non-coding (lncRNAs) and other types of ncRNAs [7,8] and are implicated in various aspects of growth, such as neuronal, muscle and germline development [9,10,11]. One such lncRNA is the growth arrest-specific 5 (*GAS5*), which was originally found to accumulate in growth-arrested cells, acting as a decoy hormone response element for the glucocorticoid receptor (GR) and hence, blocking the upregulation of gene expression by activated GR [12,13,14] (Figure 1). *GAS5* has a 5′ upstream oligopyrimidine tract sequence (5′TOP class genes) [15,16]. Serum starvation or treatment with inhibitors of protein translation can attenuate the translation of these 5′TOP RNAs and affect their degradation [17], leading to high numbers of spliced, mature *GAS5* RNA molecules [16].

**Figure 2:**
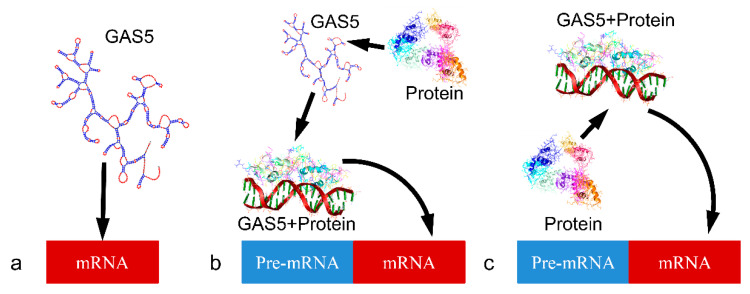
The three modes of action of *GAS5*. (**b**) *GAS5* acts directly on gene expression by regulating translation. (**a**) *GAS5* acts indirectly on gene expression. This includes the formation of a *GAS5*/protein complex regulating gene expression on the transcription level and finally again (**c**) indirectly through the formation of a *GAS5*/protein complex, which acts as a post-transcriptional regulatory mechanism. (The diagram was adopted from [20]. Exemplary molecules presented include the crystal structure of the DNA-free Glucocorticoid Receptor DNA Binding Domain with reference no. 6CFN [21], which is depicted as “Protein” and the *GAS5*/Protein complex is the crystallographic analysis of the interaction of the glucocorticoid receptor with DNA with reference no. 1R4R [22].)

The regulation of the GR function is a complicated process, still unknown to a great extent. The role of *GAS5* was previously reported in childhood obesity, where it was shown to act as a regulatory repressor element of the GR [13]. In addition, *GAS5* was shown to play a role in metabolic processes such as obesity, anorexia or overweight situations. Interestingly, its expression in in vivo samples was influenced by methylation differences on its promoter region. If metabolic disorders are affected by gene expression and regulation, then, in the short-term, this should be evident in GR-related genes and regulatory elements such as *GAS5*. On the other hand, in the long-term perspective, metabolic disorders could be reflected in premature events, and be marked on the genome as methylated genes, as for example in infancy. These findings indicate that *GAS5* manifests multifaceted roles in various physiological processes, including tumor ontogenesis [19]. *GAS5* acts as a gene regulatory element through three basic modes of action. The first refers to its direct connection to its target gene, post-transcriptionally (Figure 2a). The second includes the indirect mechanism, which involves the binding of *GAS5* with a regulatory protein (e.g., GR) and the subsequent regulation of gene transcription (Figure 2b). There is also a secondary, indirect mode of action, which involves the formation of a *GAS5*/protein complex further acting as a regulatory element for the transcribed gene (Figure 2c).

The region of *GAS5* being responsible for binding to the GR and hence, crucial for its transcriptional repression, is enclosed between nucleotides 400 and 598. This portion of human *GAS5* contains two glucocorticoid response elements (GREs) at nucleotides 539–544 (GRE-1) and 553–559 (GRE-2), which double back and complement each other with a hairpin structure [23,24] (Figure 1a). These same GRE elements are preserved in mouse Gas5, which is the only other Gas5 sequence available, although they share ~70% nucleotide homology in their exonic sequences [15,16]. In addition, a “mineralocorticoid response element” (MRE) is found at nucleotides 473–478, but it does not have a perfectly complementing sequence and, therefore, cannot form double-stranded RNA.

*GAS5* shares common responsive sequences with other steroid receptors, such as the mineralocorticoid (MR), progesterone (PR) and androgen (AR) receptors, and can thus suppress their transcriptional activity in a ligand-dependent fashion. On the other hand, *GAS5* does not affect the transcriptional activity of the peroxisome proliferator activating receptor δ (PPARδ) or p53. Likewise, *GAS5* functions as a general co-repressor of some steroid hormone receptors, repressing their transcriptional activity by binding to their DNA binding domain, as in the case of the transactivation domain (TAD) of VP16 fused with the DNA binding domain of GAL4 [13,25]. However, there is no single answer to the molecular mechanism of *GAS5* action, as differences have been found between rodent Gas5 and human *GAS5* functions [26].

## 3. *GAS5* in Tumor Therapy-Related Resistance

The role of *GAS5* in cancer ontogenesis and progression is a relatively new subject of investigation. Yet, the role of *GAS5* in therapy-induced resistance observed across different types of tumors is important and needs to be further understood [13,16,27,28,29].

### 3.1. GAS5 in Leukemia

**Figure 3:**
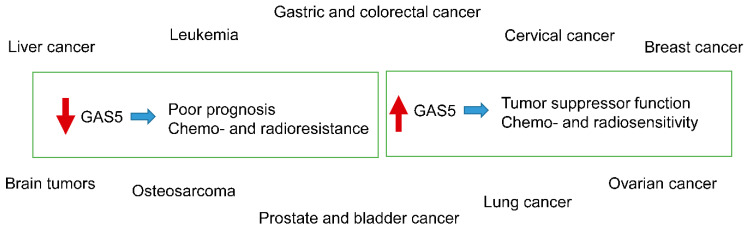
The action of *GAS5* on different tumor types. *GAS5* down-regulation or mutations are related to poor prognosis, as well as chemo- and radioresistance. On the other hand, the upregulated *GAS5* functions as a tumor suppressor and is related to chemo- and radiosensitivity.

*GAS5* expression was recently found to be tightly linked to therapy progression in acute lymphoblastic leukemia (ALL) [30]. In the study of Gasic et al. [30], *GAS5* expression was reduced at day 33 of the induction therapy as compared to day 15, yet with still higher levels, compared to the time of diagnosis. This report suggested two interesting findings. The first was that *GAS5* expression was elevated due to treatment and the second, *GAS5* expression was low at diagnosis. At the same time, a recently discovered polymorphism in *GAS5* was found to be linked with poor prognosis in acute myeloid leukemia (AML) patients [31]. The interesting finding was that *GAS5* molecules without the polymorphism, rs55829688 CC, were found to manifest higher expression levels in peripheral blood cells, compared to those that bared the polymorphism, rs55829688T [31]. However, in another report, it was shown that the down-regulation of *GAS5* led to the rescue of primary and malignant T-lymphocytes from the inhibition of the mammalian target of rapamycin (mTOR) [32]. In particular, this study showed that *GAS5* has tumor suppressor activity since it could suppress tumor growth, while, when silenced, tumor cells recovered and increased their proliferation rate [32] (Figure 3).

### 3.2. GAS5 in Cervical Cancer

Cervical cancer ranks second in women and is the fourth leading cause of deaths related to cancers. It can be very aggressive and, as such, it is still the subject of intense research. There are few reports regarding *GAS5* in cervical cancer. A recent study found that *GAS5* interacts with miR-106b and this complex inhibits the expression of the immediate early response 3 gene (IER3), leading to sensitivity to radiation therapy [33]. In another report, *GAS5* over-expression was shown to be connected to the down-regulation of miR-21 and the subsequent phosphorylation of STAT3 and E2F3 [33]. *GAS5* over-expression can also reduce the expression of two miR-21 targets: *TIMP3* and *PDCD4* [33]. All these events have been observed to lead to a G_0_/G_1_ arrest and enhancement of cisplatin-induced apoptosis [33]. Similarly, another study was in agreement with the findings by Gao et al. (2019), suggesting that *GAS5* negatively regulates miR-21 and upheaves cisplatin resistance [34] (Figure 3).

### 3.3. GAS5 in Breast Cancer

Breast cancer is the most common type of malignancy in women and a leading cause of death. It is a complex, heterogeneous disease classified into hormone-receptor-positive, human epidermal growth factor receptor-2 overexpressing (HER2+) and triple-negative breast cancer (TNBC) based on histological features [35]. Although early diagnosis is of paramount importance for the treatment and prognosis of this tumor, there is still a lot to understand on the mechanisms of action of *GAS5* in it [36]. Overall, *GAS5* is also considered to function as a tumor suppressor in breast cancer [27,37,38,39]. This was also reported in a recent work, which indicated that *GAS5* is down-regulated in breast cancer and that it negatively impacts disease prognosis [33]. A way to alleviate *GAS5* down-regulation was proposed via the inhibition of the mTOR signaling pathway [40]. Interestingly, the magnitude of cell death, in vitro, was directly proportional to *GAS5* expression levels [40]. Finally, *GAS5* was able to promote apoptosis in estrogen receptor (ER)-positive cells and in the case of *GAS5* silenced cells, the inhibition of the PI3K/mTOR signaling pathway was able to recover *GAS5* expression [40]. This finding was quite interesting, because it postulated that in the case of low levels of *GAS5* expression, the inhibition of the mTOR pathway could be a complementary therapeutic target in the treatment of breast cancer. Furthermore, *GAS5* has been linked to trastuzumab resistance, which is a main obstacle in HER2-positive breast cancer cells [41]. Li et al. (2016), showed that the down-regulation of *GAS5* is partly responsible for trastuzumab and lapatinib resistance. Both drugs interrupt the HER2/neu and EGFR pathways. In agreement with previous studies, it becomes evident that *GAS5* acts as tumor suppressor by interacting with miR-21 [41]. Tamoxifen is one of the basic chemotherapeutic agents in the treatment of breast cancer. In a recent report, it was shown that the down-regulation of *GAS5* is related to tamoxifen resistance. In particular, it was found that *GAS5* functions as a sponge for miR-222 suppressing PTEN expression and thus, inhibiting tamoxifen resistance. On the contrary, *GAS5* down-regulation functions reversely and tamoxifen resistance is promoted [42]. These findings are in agreement with a recent report where it was shown that *GAS5* is down-regulated in breast cancer tissues and linked to chemotherapy resistance [43]. Consequently, it seems that *GAS5* can be considered as new player in cancer ontogenesis, progression and prognosis, as well as it may have prognostic and therapeutic applications in this disease. Several new drugs have been designed and synthesized for the treatment of breast cancer (Figure 3).

### 3.4. GAS5 in Ovarian Cancer

Ovarian cancer is a grave gynecological tumor and there are not many studies concerning the role of *GAS5* in it or its association with chemoresistance in this tumor. However, similar to other gynecological tumors, it seems that all studies converge to the conclusion that *GAS5* acts as a tumor suppressor in ovarian cancer, as well [44,45,46,47]. In a recent study, *GAS5* was found to be down-regulated in this disease [41]. In particular, in a meta-analysis of 561 microarrays and 136 RNA-seq specimens, *GAS5* was down-regulated and manifested high sensitivity and specificity in predicting platinum-based chemoresistance [48]. *GAS5* was also found to be down-regulated in epithelial ovarian cancer in another study, where it was related to disease prognosis, in particular [49]. At the same time, *GAS5* was found to be down-regulated in cisplatin resistant tumors. On the contrary, its up-regulation had the opposite effect, which significantly enhanced the sensitivity of ovarian cancer cells to cisplatin, both in vivo and in vitro. Further on, the up-regulation of *GAS5* was found to increase both the ratio of G_0_/G_1_ arrest and apoptosis in ovarian cancer [42]. The same study reported that a probable mechanism for *GAS5* action was mediated through the regulation of *PARP1* by recruiting the transcription factor E2F4 to its promoter and subsequent MAPK pathway activity [50] (Figure 3).

### 3.5. GAS5 in Prostate and Bladder Cancers

Both prostate [51] and bladder cancers [52] are considered to be two major tumor types and causes of cancer-related deaths. In prostate cancer, *GAS5* was found to be down-regulated, while it was down-regulated in radio-resistant prostate tumor cells [53]. This effect, was found to be alleviated by the addition of a-Solanine, which up-regulates *GAS5* and at the same time, confers sensitivity to radiotherapy [53]. Similarly, *GAS5* was found to be down-regulated in transitional cell carcinomas of the urinary bladder, and its down-regulation was found to be positively correlated with higher pathological grades of the tumor [54]. However, in an in vitro system, *GAS5* overexpression could reduce chemo-resistance to doxorubicin and promoted apoptosis [54] (Figure 3).

### 3.6. GAS5 in Lung Cancer

Lung cancer is the most common cause of death from tumor-related diseases [55]. A recent study reported that *GAS5* is down-regulated in lung cancer cells and at the same time, its knockdown increased cis-platin IC50 in an in vitro system, while its overexpression decreased it [56]. In the same study, it was found that *GAS5* knockdown resulted in decreased autophagy in vitro, and therefore, resistance to cis-platin [56]. Similarly, *GAS5* up-regulation was found to be a significant factor of inhibition of tumorigenesis and an enhancer of radiosensitivity [57]. In addition, the mechanism of enhancement of radiosensitivity was found to function via the suppression of miR-135b in non-small cell lung cancer cells [57]. Another recent study confirmed that *GAS5* plays a significant role in non-small cell lung cancer, participating in cis-platin resistance. Cao et al. reported that chemo-sensitivity is modulated by the tumor suppressor PTEN [58]. In the same study, it was found that a significant low *GAS5* expression in non-small cell lung cancer patients was correlated with poorer prognosis. In an in vitro system, *GAS5* knockdown promoted cell viability and regulated chemo-sensitivity to cis-platin. The authors showed that *GAS5* competed with PTEN for miR-21 binding, indicating a strong evidence that *GAS5*/miR-21/PTEN interactions are significant in cis-platin sensitivity in non-small cell lung cancer cells [58]. Similarly, *GAS5* was found to bind miR-21 and miR-23a, at the same time up-regulating PTEN and inhibiting PI3K/Akt phosphorylation [59]. This mechanism was found to function as an angiogenesis inhibitor, signifying that *GAS5* could be targeted therapeutically in order to inhibit angiogenesis in non-small cell lung cancer [59] (Figure 3).

### 3.7. GAS5 in Gastric and Colorectal Cancers

Gastric cancer is the fourth most common malignancy, and the second most common cause of cancer-related deaths in the world [60]. *GAS5* also plays a significant role as a tumor suppressor in gastric cancer [61,62,63,64]. A recent study highlighted the fact that *GAS5* expression was significantly down-regulated in gastric cancer tissues, and that it was down-regulated in adriamycin-resistant cells [65]. *GAS5* was also found to have higher levels of promoter methylation in SGC-7901 cells, conferring resistance to chemotherapy [65]. There are no reports on the role of *GAS5* in chemoresistance in colorectal cancer; yet, reports suggest that *GAS5* is responsible for tumor suppression, inhibition of proliferation, metastasis and invasion [66]. In addition, a recent study indicated that *GAS5* inhibits angiogenesis and metastasis in colorectal cancer by suppressing the Wnt/beta-catenin signaling pathway, which is dedicated to promoting cell invasion and migration in this type of tumor (Figure 3) [67]. Recently, a 5-bp indel polymorphism (rs145204276) was found in the *GAS5* promoter region and proposed to have a carcinogenic effect [68].

### 3.8. GAS5 in Liver Cancer

Liver cancer, in particular, hepatocellular carcinoma, is predominately present in eastern Asia and its rates are increasing in the northern hemisphere [69]. Liver cancer has a very fast progressing time span posing a significant threat to life. There are no reports on the role of *GAS5* in chemosensitivity or chemoresistance in liver cancer. Yet, there are some interesting reports suggesting that *GAS5* plays a synergistic role in the anti-tumor action of flavonoids and phytochemicals. In particular, phytochemicals, such as curcumin, resveratrol, sulforaphane, berberine and gambogic acid, have all been examined for their connection with non-coding RNAs. *GAS5* was reported as one of the ncRNAs that is regulated by phytochemicals, which can synergistically affect tumor development and progression. When phytochemicals were administered in combination with chemotherapeutics, they were found to have an additive effect on the overexpression of *GAS5* and the sensitization of cancer cells to chemotherapy. Finally, a recent study showed that corylin, a flavonoid extracted from the plant *Psoralea corylifolia* L. (Fabaceae), suppresses tumor growth and progression [70]. The interesting finding was that corylin was found to exert such effects on tumor growth through activation of *GAS5* [70] (Figure 3).

### 3.9. GAS5 in Brain Tumors

Brain tumors, or tumors of the central nervous system, along with their extreme diversity, present a special case of malignancy due to the anatomical position in which they are diagnosed. This point is further strengthened by the fact that in several tumors, either benign or extremely aggressive, surgical excision is a drastic solution towards therapy, while in the case of brain tumors, this is not always the case, or it is less feasible. There are no studies connecting *GAS5* to chemoresistance. In fact, there are very few studies on the role of *GAS5* in brain tumors, in general. Yet, all the present studies agree that *GAS5* functions as a tumor suppressor and inhibits tumor proliferation, invasion, metastasis and migration [71,72,73,74,75] (Figure 3).

### 3.10. GAS5 in Osteosarcoma

Another tumor type that we investigated is osteosarcoma. Osteosarcoma is a rare malignancy of the childhood with an incidence of 4–5 new cases per million per year [76]. It is an aggressive malignant neoplasm that arises from primitive transformed cells of mesenchymal origin, exhibits osteoblastic differentiation and produces malignant osteoid [77]. There are no reports concerning the role of *GAS5* in osteosarcoma with respect to chemotherapy-related resistance. However, there are some reports referring to *GAS5* as a significant gene in the tumor’s progression. In particular, miR-663a and *ZBTB7A* were found to protect osteosarcoma from endoplasmic reticulum stress-induced apoptosis, through the down-regulation of *GAS5* [78], while in a similar study the CtBP1-HDAC1/2-IRF1 transcriptional complex was also found to be down-regulated in osteosarcoma cells [79]. In addition, it was found that *GAS5* sponges miR-203a [80] and miR-221 [81], thus suppressing tumor growth and inhibiting tumor invasion (Figure 3).

## 4. The Special Case of *GAS5* and miRNAs

### 4.1. GAS5 and miRNAs in Leukemia

The topic of *GAS5* and miRNAs could not escape the attention of the present work. There are very few reports on the connection between *GAS5* and miRNAs. In the case of leukemia, there is one report suggesting the interaction of *GAS5* with miR-222, since their expression is negatively correlated [82]. In this study, it was also found that *GAS5* over-expression was related to the inhibition of leukemic cells proliferation, the enhancement of leukemic cell apoptosis and the inhibition of tumor cell invasion [82] (Table 1).

### 4.2. GAS5 and miRNAs in Cervical Cancer

Similarly, few reports are available on the connection of *GAS5* and miRNAs in cervical cancer. Yet, all studies agree that *GAS5* acts as a suppressor or “sponge” for oncogenic miRNAs, whereas its overexpression is closely related to tumor suppression and induction of therapy-related sensitivity. In particular, previous studies indicated that *GAS5* interacts with miR-222 [82], miR-106b [33], miR-135a [83], miR-21 [84] and miR-205 [34,85], conferring tumor suppressor properties and induction of sensitivity to chemo- and radiotherapy (Table 1).

### 4.3. GAS5 and miRNAs in Breast Cancer

The association between *GAS5* and miRNAs has been widely studied in breast cancer. A recent study highlighted the role of *GAS5* in breast cancer and adriamycin resistance, through the gene’s interaction with miR-221-3p [86]. Another report showed that *GAS5* manifested tumor suppressor effects and induced chemosensitivity to breast cancer cells by indirectly targeting the miR-378-5p/SUFU signaling pathway [87], as well as by competitively binding miR-196a-5p [87]. Additionally, *GAS5* appeared to be a direct target of miR-221/222, suppressing tumor proliferation and enhancing tumor cell apoptosis [88]. In another report, it was shown that *GAS5* stimulates autophagy through the miR-23a/ATG3 axis, where it acts as a miRNA sponge [89]. Interestingly, miR-21 also has an oncogenic role in breast cancer, where it induces chemo- and radiosensitivity [41] (Table 1).

### 4.4. GAS5 and miRNAs in Ovarian Cancer

Although ovarian cancer is very common in women, there is only one report investigating the association between miRNAs and *GAS5* in it. In this, the role of miR-196-5p in relation to *GAS5* was reported. *GAS5* down-regulation was found to be related to high miR-196-5p expression, which induced tumor cell proliferation and progression. Thus, *GAS5* up-regulation confers tumor cell proliferation inhibition [46] (Table 1).

### 4.5. GAS5 and miRNAs in Prostate and Bladder Cancers

In the case of prostate and bladder cancers, two reports highlighted the connection of *GAS5* with miRNAs. In particular, it was reported that *GAS5* is down-regulated due to its targeting from miR-940 [91]. The relation between *GAS5* and miR-940 was reported to be a possible prognostic factor. Finally, a recent study indicated that *GAS5* negatively regulates miR-18a and, thus, confers radiosensitivity in human prostate cells [53] (Table 1).

### 4.6. GAS5 and miRNAs in Lung Cancer

Several reports have also identified the connection between *GAS5* and miRNAs. A recent report showed that *GAS5* probably indirectly regulates miR-21, whereas its over-expression suppresses miR-21 expression and, hence, increases radiosensitivity of lung tumor cells [92]. Recently, it was also shown that miR-29-3p antagonizes *GAS5* for binding PTEN [59]. It was also reported that *GAS5* exosomes are the basic vehicle of transmission conferring tumor inhibition [59]. The connection of *GAS5*/PTEN and miRNAs is also stated to be of significance through the competitive binding with miR-21 [58]. Similarly, the role of *GAS5*/PTEN is also shown to be of significance in lung cell proliferation and metastasis in connection to miR-205 [93]. Another recent study suggested that *GAS5* directly binds and suppresses miR-135b, enhancing radiosensitivity [57]. Finally, a connection between *GAS5* and miR-23a has been reported, where miR-23a was found to suppress *GAS5* expression and enhance tumor cell proliferation and tumorigenesis [94] (Table 1).

### 4.7. GAS5 and miRNAs in Gastric and Colorectal Cancers

In gastric cancer, three miRNAs have been reported to relate to *GAS5*, miR-18a [95], miR-106a-5p [63] and miR-222 [96]. In the case of miR-18a, it was reported that *GAS5* directly binds to it, inhibiting tumor growth via the stimulation of the activity of natural killer (NK) cells [95]. On the other hand, *GAS5* functions as sponge for miR-106a-5p, inactivating the Akt/mTOR pathway [63]. Finally, miR-222 was reported to directly bind to *GAS5* similarly, as in all previous cases, suppressing tumor cell proliferation [96]. In colorectal cancer, two different miRNAs were reported, miR-182-5p [97] and miR-221 [98]. *GAS5* could directly bind to miR-182-5p and inhibit tumor cell proliferation through the miR-182-5p/FOXO3a axis [97]. Similarly, miR-221 is negatively regulated to *GAS5* expression. If overexpressed, *GAS5* can suppress miR-221 expression and subsequently inhibit tumor cell proliferation in colorectal cancer [98] (Table 1).

### 4.8. GAS5 and miRNAs in Liver Cancer

In the case of gastric cancer, five miRNAs have been reported to be related to *GAS5*, miR-222 [99], miR-21 [100,101], miR-544 [102], miR-135b [103] and miR-34a [104]. *GAS5* was shown to sensitize hepatocellular cancer cells to chemotherapy by sponging miR-222 [99]. Similarly, *GAS5* directly acts as a sponge for miR-21, suppressing its expression and subsequently inhibiting hepatocellular carcinoma proliferation [100,101]. In the case of miR-544, *GAS5* negatively regulates its expression, inhibiting tumor cell proliferation [102]. *GAS5* inhibits cell proliferation also through the miR-544/RUNX3 pathway [102], where it stimulates NK cell activity and inhibits tumor growth [95]. In addition, *GAS5* and miR-135b reversely correlated and as reported in other tumors, *GAS5* over-expression reduces miR-135b expression and, thus, inhibits tumor cell proliferation [103]. Finally, miR-34a manifested a different mode of action with respect to *GAS5*. It appeared that *GAS5* and miR-34a were positively correlated in three types of tumors; in hepatocellular carcinoma, glioblastoma and renal cell carcinoma [104]. *GAS5* under-expression was also related to tumor progression and proliferation (Table 1).

### 4.9. GAS5 and miRNAs in Brain Tumors

Since brain tumors are not easily manageable, there are not many reports on the connection of *GAS5* and miRNAs. The existent studies are concerned with reports on glioma. In particular, two miRNAs are found to be related to *GAS5*. The miR-106b-5p [105] and miR-18a-5p [73]. Both of them were found to be significantly up-regulated in glioma cells, while *GAS5* was down-regulated. Additionally, it was found that *GAS5* over-expression results in miRNA down-regulation (Table 1).

### 4.10. GAS5 and miRNAs in Osteosarcoma

Three miRNAs are related to *GAS5* in osteosarcoma: miR-663a [79], mIR-203a [80] and miR-221 [81]. The miR-663a indirectly suppresses *GAS5* through the inhibition of its target, ZBTB7A [79]. Moreover, miR-203a suppresses *GAS5*, deactivates TIMP2, but activates the PI3K/AKT/GSK2β pathway with simultaneous inhibition of the NF-κB signaling cascade [80]. Therefore, *GAS5* indirectly regulates miR-203a, as also supported by their reverse-correlated expression. Finally, *GAS5* can directly suppress miR-221 through the miR-221/ARH1 pathway [81] (Table 1).

## 5. Discussion

Functional ncRNAs affect every aspect of the biology in many organisms, from bacteria to higher eukaryotes. Specifically, they affect all stages of the coding sequence, including mRNA transcription, degradation and translation, and/or the nuclear translocation of proteins [5,106,107]. Among them, *GAS5* is mechanistically related to the bacterial 6S RNA, which binds the RNA polymerase and inhibits transcription [107,108]. Regarding nuclear receptor related ncRNAs, the ncRNA coactivator steroid receptor RNA activator (SRA) enhances nuclear receptor-induced transcriptional activity by associating with cofactor proteins, its stem-loop interacting protein, called SLIRP, and a pseudo-uridine synthase Pus1p [109,110]. *GAS5* is distinct from SRA in its activity and mode of action, while, similarly to SRA and other ncRNAs, its interaction with regulatory proteins might be critical for Gas5-mediated suppression of GR-induced transcriptional activity. Indeed, in relation to complex transcriptional regulation of endogenous, chromatin-associated genes [49], it would be interesting to investigate if *GAS5* can mimic the conformation of chromatin-integrated DNA interaction with histone-bound proteins and/or other chromatin components, with which the GR normally interacts to stimulate the transcription of endogenous, glucocorticoid-responsive genes.

In the present study, we explored the expression of *GAS5* along with that of various miRNAs across different tumor types and focused on its role in therapy-related sensitivity to these cells. The main conclusion is that *GAS5* seems to exert a tumor-suppressive role in the process of carcinogenesis across all tumor types. It does so, by interacting with or modulating the expression of various gene targets. As such, *GAS5* participates in tumor growth, proliferation, invasion, metastasis inhibition, as well as the induction of apoptosis. However, it seems that *GAS5* is also involved in the therapeutic response of cancer patients. Here, we review both in vitro and in vivo studies showing that *GAS5* contributes to the sensitization of cancer cells to chemotherapy and radiotherapy [33]. The tumor suppressive role of *GAS5* was recently supported by others as well [111,112,113], and all clues suggest that *GAS5* could be used a promising biomarker for disease diagnosis, tumor progression, or even as a therapeutic marker. However, there are a few studies investigating in-depth the role of *GAS5* in human tumors. Apart from differential expression, diverse genetic variants within *GAS5* have also been proposed to affect drug response, and could, thus, facilitate the categorization and dose adjustment [111].

## 6. Conclusions

Several studies highlight that *GAS5* plays an important role in various pathological and physiological conditions. Overall, *GAS5* acts as a tumor suppressor, whose down-regulation is directly connected to tumor progression, tumor cell proliferation and therapy-related resistance across different types of tumors. The agreement of different studies on the role of *GAS5* makes it a new attractive target for the prognosis and therapy of different cancer types.

## Figures and Tables

**Table 1:** Summary of the relation of *GAS5* and miRNAs in several tumors.

Tumor	miRNA	Relation between *GAS5* and miRNA	*GAS5* Effect on Tumor	Effect on Therapy-Related Resistance	Citation
Leukemia	miR-222	Direct Suppression	Tumor suppressor	Unknown	Jing et al. (2019) [82]
Cervical Cancer	miR-106b	Direct Suppression/Sponge	Tumor suppressor	Induces chemo- and radiosensitivity	Gao et al. (2019) [33]
	miR-135a	Direct Suppression	Tumor suppressor	Induces chemo- and radiosensitivity	Yan et al. (2020) [83]
	miR-21	Direct Suppression	Tumor suppressor	Induces chemo- and radiosensitivity	Yao et al. (2019), Li (2016) [41,84]
	miR-205	Direct Suppression	Tumor suppressor	Induces chemo- and radiosensitivity	Yang et al. (2017), Wen et al. (2017) [34,85]
Breast Cancer	miR-221-3p	Direct Suppression/Sponge	Tumor suppressor	Induces chemo- and radiosensitivity	Chen et al. (2020) [86]
	miR-378-5p	Indirect Suppression/Sponge	Tumor suppressor	Induces chemo- and radiosensitivity	Zheng et al. (2020) [87]
	miR-221/222	Direct Suppression	Tumor suppressor	Induces chemo- and radiosensitivity	Zong et al. (2019), Gu et al. (2018) [42,88]
	miR-23a	Direct Suppression/sponge	Tumor suppressor/induces autophagy	Unknown	Gu et al. (2018) [89]
	miR-196a-5p	Direct Suppression/sponge	Induces autophagy	Unknown	Li et al. (2018) [90]
	miR-196a-5p	Direct Suppression	Tumor suppressor	Induces chemo- and radiosensitivity	Zheng et al. (2020) [87]
	miR-21	Direct Suppression/Sponge	Tumor suppressor	Induces chemo- and radiosensitivity	Li (2016) [41]
Ovarian Cancer	miR-196a-5p	Direct Suppression	Tumor suppressor	Induces chemo- and radiosensitivity	Zhao et al. (2018) [46]
Prostate Cancer	miR-940	Indirect Suppression	Tumor suppressor	Unknown	Chen et al. (2017) [91]
	miR-18a	Indirect Suppression	Tumor suppressor	Induces chemo- and radiosensitivity	Yang et al. (2019) [53]
Lung Cancer	miR-21	Indirect Suppression	Tumor suppressor	Induces chemo- and radiosensitivity	Chen et al. (2020) [92]
	miR-29-3p	Indirect Suppression	Tumor suppressor	Induces chemo- and radiosensitivity	Cheng et al. (2019) [59]
	miR-21	Indirect Suppression	Tumor suppressor	Induces chemo- and radiosensitivity	Cao et al. (2017) [58]
	miR-205	Direct Suppression	Tumor suppressor	Unknown	Dong et al. (2019) [93]
	miR-135b	Direct Suppression	Tumor suppressor	Induces chemo- and radiosensitivity	Xue et al. (2017) [57]
	miR-23a	Indirect Suppression	Tumor suppressor	Unknown	Mei et al. (2017) [94]
Gastric Cancer	miR-18a	Direct Suppression	Tumor suppressor	Unknown	Wei et al. (2020) [95]
	miR-106a-5p	Indirect Suppression	Tumor suppressor	Unknown	Dong et al. (2019) [63]
	miR-222	Direct Suppression/sponge	Tumor suppressor	Unknown	Li et al. (2017) [96]
Colorectal Cancer	miR-182-5p	Direct Suppression/sponge	Tumor suppressor	Induces chemo- and radiosensitivity	Cheng et al. (2018) [97]
	miR-221	Indirect Suppression	Tumor suppressor	Unknown	Liu et al. (2018) [98]
Liver Cancer	miR-222	Direct Suppression/sponge	Tumor suppressor	Induces chemo- and radiosensitivity	Zhao et al. (2020) [99]
	miR-21	Direct Suppression/sponge	Tumor suppressor	Unknown	Wang et al. (2018), Hu et al. (2016) [100,101]
	miR-544	Indirect Suppression	Tumor suppressor	Unknown	Fang et al. (2019) [102]
	miR-135b	Indirect Suppression	Tumor suppressor	Unknown	Yang et al. (2019) [103]
	miR-34a	Indirect Suppression	Tumor suppressor/sponge	Unknown	Toraih et al. (2018) [104]
Glioma	miR-106b	Indirect Suppression	Tumor suppressor/sponge	Unknown	Huang et al. (2020) [105]
	miR-18a-3p	Indirect Suppression	Tumor suppressor/sponge	Unknown	Liu et al. (2018) [73]
Osteo-sarcoma	miR-663a	Indirect Suppression	Tumor suppressor/sponge	Unknown	Zhang et al. (2019) [79]
	miR-203a	Indirect Suppression	Tumor suppressor/sponge	Unknown	Wang et al. (2018) [80]
	miR-221	Direct Suppression	Tumor suppressor/sponge	Unknown	Ye et al. (2017) [81]

## Abbreviations

ALL	Acute Lymphoblastic Leukemia
AML	Acute Myeloid Leukemia
AR	Androgen Receptor
*GAS5*	Growth Arrest Specific 5
GR	Glucocorticoid Receptor
GRE	Glucocorticoid Response Element
lncRNAs	long non-coding RNAs
miRNA	microRNA
MR	Mineralocorticoid Receptor
MRE	Mineralocorticoid Response Element
ncRNA	non-coding RNA
NK	Natural Killer cells
piRNAs	piwi-interacting
PR	Progesterone Receptor
snoRNAs	small non-coding nucleolar RNAs
SRA	Steroid Receptor RNA Activator
TAD	Transactivation Domain
